# Reconciling the IPC and Two-Hit Models: Dissecting the Underlying Cellular and Molecular Mechanisms of Two Seemingly Opposing Frameworks

**DOI:** 10.1155/2015/697193

**Published:** 2015-12-06

**Authors:** Carlos F. M. Morris, Muhammad Tahir, Samina Arshid, Mariana S. Castro, Wagner Fontes

**Affiliations:** ^1^Laboratory of Biochemistry and Protein Chemistry, Department of Cell Biology, Institute of Biology, University of Brasilia, 70910-900 Brasilia, DF, Brazil; ^2^Laboratory of Surgical Physiopathology (LIM-62), Faculty of Medicine, University of Sao Paulo, 01246-904 Sao Paulo, SP, Brazil

## Abstract

Inflammatory cascades and mechanisms are ubiquitous during host responses to various types of insult. Biological models and interventional strategies have been devised as an effort to better understand and modulate inflammation-driven injuries. Amongst those the two-hit model stands as a plausible and intuitive framework that explains some of the most frequent clinical outcomes seen in injuries like trauma and sepsis. This model states that a first hit serves as a priming event upon which sequential insults can build on, culminating on maladaptive inflammatory responses. On a different front, ischemic preconditioning (IPC) has risen to light as a readily applicable tool for modulating the inflammatory response to ischemia and reperfusion. The idea is that mild ischemic insults, either remote or local, can cause organs and tissues to be more resilient to further ischemic insults. This seemingly contradictory role that the two models attribute to a first inflammatory hit, as priming in the former and protective in the latter, has set these two theories on opposing corners of the literature. The present review tries to reconcile both models by showing that, rather than debunking each other, each framework offers unique insights in understanding and modulating inflammation-related injuries.

## 1. Introduction

Many models have been put forward as an attempt to explain and counteract the real-life outcomes of several different inflammatory events in which neutrophil leukocytes play an outstanding role. Trauma, infection, hemorrhage, the response to both elective and emergency surgical interventions, and other pathological processes are incredibly prevalent in the human population. Such conditions are often complicated by nefarious immune responses that arise from these events that are, at least partially, mediated by neutrophils [1–4], since these are cells known for their central role in the mechanisms of inflammation in mammals [5, 6].

Logically, there is an ongoing effort to explain the inflammatory dynamics arising from these types of insults, as a first step in the direction of modulating and perhaps coordinating such responses in order to improve outcomes, reduce hospitalization times, and even prevent death.

The two-hit or multiple-hit hypothesis is a model that explains how sequential insults can synergically contribute to an inappropriate immune response [7] in which MODS/MOF (multiple organ dysfunction syndrome/multiple organ failure) is often the endpoint. As a broad definition, the two-hit model hypothesizes that an initial inflammation-triggering event, such as pancreatitis, trauma, burns, excessive bleeding, or elective surgery, can set in motion a priming condition for the immune system that can cause limited expression of SIRS (systemic inflammatory response syndrome) or other mild effects if left alone. Additional hits or insults (e.g., second-look laparotomy, infection, further blood loss, or ischemic injury during the process of aneurysm repair) are capable of causing an extraordinary and exaggerated immune response [8] that can evolve to MODS/MOF and death.

Ischemic preconditioning or IPC, on the other hand, does not represent an actual attempt to explain the inflammatory processes involved in SIRS/sepsis and its continuum of MODS/MOF. Rather, it is a collection of techniques that make use of the dynamics of the inflammatory response to generate a modulatory effect over these events. IPC is a demonstrable, observable, reproducible phenomenon in which a nonlethal, mild, and often cyclic ischemic event has the capacity to protect organs and tissues from a secondary, prolonged, and otherwise deleterious ischemic event [9], mitigating the response to ischemia and the ischemia-reperfusion injury (IRI).

Incidentally, the predominance of information in the literature about both models has a marked timeline difference. Throughout the late 1980s and early 1990s the two-hit model was considered a good standard as an intuitive and empirical explanation for some real-life chronologically based events seen in trauma and septic patients. Conversely, while the manipulation of the inflammatory response through exposure to controlled ischemic scenarios was already underway in the late 1980s [10], it was not until recently that IPC was shown to have clinical and surgical applications that far surpass those initially conceived and has, not without merit, been increasingly present in the literature.

The shift in the literature and the seemingly obvious difference as to how the two models treat a first inflammatory event, on one hand as a first/priming hit, and on the other hand as a protective/beneficial insult, have led to the notion that one model is capable of debunking the previous theory. In the following pages, we shed some light on some of the paradoxes regarding the coexistence of both models and try to reconcile both theories as simultaneously valid answers to some very different questions.

## 2. Two-Hit Model: An Intuitive Explanation for Empirical, Readily Observable Conditions

Sometime by the late 1990s the two-hit model soared to a unique position as a theory that successfully explained and accounted for many of the bedside events that accompanied trauma patients, which by the nature of their injuries were often exposed to sequential insults [11]. Another relevant well-known real-life example of the application of the two-hit model is the correction of ruptured aortic aneurysms [12], which requires imposing a second long-duration dry ischemic event for the actual repair of the initial naturally occurring hemorrhagic injury [13]. A host of experimental models has also been developed to mimic the events of multiple or sequential hits in order to further understand the processes involved in the augmentation of the inflammatory response. One example of those models is from the researchers that in 1998 demonstrated that neutrophil recruitment to the lung was increased when hemorrhagic shock (first hit) was followed by inoculation of LPS (second hit) if compared to a single-hit process [14]. Another group, on the following year, demonstrated the same marked increase in PMN recruitment to the lung when subjects were exposed to a second hit with direct lung injury from LPS and immune complexes after a septic event that served as the first hit [15]. Nevertheless, examples of multiple-hit models that failed to induce the anticipated augmented immunological response can also be found in the literature. For instance, an American study from 2000 in which subjects were exposed to intratracheal injection of acid with or without previous induction of sepsis by CLP (cecal ligation and puncture) could not demonstrate a synergistic or even additive effect on the inflammatory response. Such study compared a two-hit insult versus a single-hit murine model, although their evaluation was limited to the number of PMN and the concentration of albumin present after BAL (bronchoalveolar lavage) [16]. This goes to show that the type and dynamics of the insult are to be considered when experimental models are designed to reproduce the inflammatory effects of multiple-hit insults.

As a rule, patients can be exposed to a variety of first-hit events, such as trauma itself or any number of hemorrhagic, ischemic, or infectious insults. During their hospital stay, patients are exposed to second, third, or further sequential events (e.g., laparotomy, fluid replacement therapy, blood transfusions, fracture repair surgeries, infection via catheter, or other sources). The mechanics behind it is that the first hit serves as a priming event that sets the patient towards the establishment of SIRS (systemic inflammatory syndrome). SIRS in itself is a fairly straightforward diagnosis, consisting in the identification of two or more of the following criteria [11]: (a) body temperature below 36°C or above 38°C; (b) heart rate higher than 90 bpm; (c) respiratory rate in excess of 20 mpm or PaCO_2_ lower than 32 mmHg; and (d) total white blood cell count above 12.000 mm^3^ or below 4.000 mm^3^ or the presence of over 10% band forms. If the first insult is by any chance infectious in nature, SIRS is loosely termed sepsis [17].

After the establishment of SIRS, a secondary, seemingly trivial, insult can jumpstart a detrimental organic response that can culminate in potentially lethal conditions such as MODS/MOF [14]. Depending on the type of sequential hits, the path following SIRS (which in this text is generally considered being end result of the first hit) is somewhat dualistic in nature. A first, anti-inflammatory state can ensue, which is called CARS (compensatory anti-inflammatory response syndrome) that, in and of itself, can be dangerous since it predisposes the body to infection that can in turn serve as one of the following hits. During CARS, immunosuppression occurs via impairment of T-cell function that can deteriorate the pathophysiological cascade and lead to infection, sepsis, MODS/MOF, and death [18–24]. A second proinflammatory state is triggered depending on the nature of the sequential events (additional hits). Major surgery, IR-like injury by fluid reperfusion in a previously hypovolemic patient, and infection by loss of gut barrier are some of the events that can serve as second or sequential hits. Cytokines and other molecular markers for both the anti- and proinflammatory states can be simultaneously found in patients facing hospitalization from several causes of SIRS/sepsis/MODS/MOF. This observation implies that these two biological conditions are not so clearly separated in real-life biological conditions but rather serve as a dynamic modulatory system that ideally keeps both the suppression and augmentation of the inflammatory response in check throughout the clinical evolution of the patient. Additional hits tend to throw this system out of balance, causing the inflammatory response and clinical presentation to escalate. Regardless of the type of insult, the two-hit model postulates that the inflammatory response to the additional challenges is generally exaggerated, since the body has already been primed by the first event, or hit. The complex molecular cascades and the serious remote injuries triggered by this proinflammatory state are responsible for the potentially fatal state of MODS/MOF [17]. The two-hit model clearly shows that severely injured or ill patients are commonly more easily susceptible to the sequential insults, which singly or cumulatively lead to their unfavorable outcomes, observation which indeed can be easily correlated to common clinical outcomes.

The molecular pathways that explain the two-hit model are incredibly complex and, to this date, there has not been a definitive flow of events identified. One can easily understand why this is the case, since the variable nature of the first and subsequent events imposes a humongous challenge to the definition of the underlying inflammatory biochemistry behind the two-hit model theory. For that reason, any discussion of the events occurring during the progression of the multiple inflammatory hits has to take into account the specifics of every single type of insult. Below we discuss some general molecular and physiological aspects underlying the two-hit or multiple-hit inflammatory events.

### 2.1. Cellular Responses

After an initial insult the immune system is affected at a cellular level as the inflammatory cells become easily susceptible and primed to any sequential stimulus/insult and are therefore further activated by a minor sequential exposure, allowing mildly injurious stimuli to synergistically set off the inflammatory machinery and cause tissue damage [25–28] both locally and remotely. The immune response induced by the first hit can be traumatic in nature and may be limited locally as in monotrauma or it may be a massive systemic immune activation as in polytrauma [18, 21, 29–31]. Different trauma-related inflammatory actors have been recently characterized among which the complement system stands out as a key mediator [20, 21, 24, 32–37]. When the complement system is activated by any of its three pathways, it plays a pivotal role in eliminating foreign pathogens by opsonization/phagocytosis (C3b, C4b) and chemotaxic attraction of leukocytes (C3a, C5a) and also directly lysing the pathogens through the membrane attack complex (MAC, C5b-9) [35, 38–40]. The anaphylatoxins like C3a and C5a recruit phagocytes and polymorphonuclear leukocytes (PMN) to the site of injury as these anaphylatoxins are strong chemoattractants for phagocytes [41] and also induce the degranulation of mast cells, basophils, and eosinophils [35, 39, 40]. It is clear from clinical and experimental studies that after trauma the complement system gets activated both locally and at the injury site, as well as systemically [42–47]. Tissue damage and cell injury cause the release of alarmins, which are non-pathogen-derived danger signals capable of activating the innate immune responses. These include annexins, heat-shock proteins (HSPs), defensins, and classical markers of tissue injury like S100 protein and high mobility group box-1 (HMGB1) nuclear protein [48, 49]. Alarmins correlate with the heterogenic innate immune inflammatory molecules and pathogen-associated molecular patterns (PAMPs), which are recognized by the immune system as foreign molecules because of their characteristic molecular pattern [50, 51]. Together, alarmins and PAMPs form a large family of damage-associated molecular patterns (DAMPs) and are recognized by immune cells that express multiligand receptors, such as Toll-like receptors (TLRs), on their surfaces. Therefore DAMPs are capable of activating innate immune responses after trauma either when the traumatic injury is a standalone event or when the traumatic event is further complicated by infection [24, 48]. It is reasonable to assume that similar triggers are present when the first and sequential hits are not traumatic in nature.

### 2.2. Molecular Responses

Whatever the cause of the insult, it is generally accepted that cytokinemia or cytokine storm is of major importance during the biological responses inside the two-hit model [12]. Cytokines are molecules of low molecular weight that are secreted by immune cells and serve as mediators for the communication between leukocytes, interlinking innate and adaptive immune responses. Traumatic tissue injuries induce the expression of proinflammatory cytokines, such as tumor necrosis factor (TNF) and interleukin- (IL-) 1*β*, IL-6, IL-8, IL-12, IL-15, and IL-18 [52–54]. In addition to other biological roles, cytokines also activate neutrophils which are key players in the early inflammatory response to trauma or other first-hit insults [55]. Neutrophils are pulled away from the circulation to the site of injury by chemotactic molecules, such as complement anaphylatoxins and chemotactic cytokines, called chemokines, most notably IL-8 [39, 56]. Several studies have tracked cytokine production during inflammatory conditions, demonstrating their primary role in tissue damage by cellular priming/activation and also in the pathophysiology of SIRS. In particular, tumor necrosis factor-*α* (TNF-*α*), IL-1*β*, IL-6, and IL-8 have been consistently present during these observations. Nevertheless inconsistent results in terms of levels of TNF-*α*, IL-1*β*, and IL-6 have prevented a definitive association between high concentration of these agents and the risk for development of MODS/MOF [57, 58]. In an ideally regulated immune response, neutrophils play an important role in the defense and repair of injured tissues. PMN priming for cytotoxicity covers a wide range of physiologic responses, like degranulation of enzymes, superoxide anion generation, lipid mediator (LTB4) and cytokine (IL-8) production, decreased selectin expression (L-selectin), enhanced integrin expression (CD11b/CD18), cellular elongation, reduced deformability, and delayed apoptosis [59–63] in addition to other cellular events such as adhesion, rolling, and ultimately diapedesis [20, 64, 65]. Neutrophil priming results from the preexposure of the cell to priming molecules like platelet activating factor (PAF), anaphylatoxin C5a, granulocyte macrophage-colony stimulating factor (GM-CSF), LTB4, substance P, IL-8, interferon, TNF-*α*, LPS, L-selectin cross-linking, and CD18 cross-linking [20, 66–68], which could arise from exposure to first-hit events [66, 69]. Some investigators have suggested that circulating monocytes and tissue macrophages also become primed after severe injury [70–72]. Despite the beneficial effects of neutrophils in host defense, a dysfunction in priming and subsequent cellular activation may result in an overwhelming inflammatory response. Such response leads to tissue injury of previously healthy sites via the local release of toxic metabolites and enzymes that may lead to acute respiratory distress syndrome (ARDS), MODS/MOF, secondary blood-brain barrier dysfunction, and brain edema after traumatic brain injury [21, 68, 73–79].

### 2.3. Vascular Responses

Aside from circulating cytokines and signaling molecules, the response of the endothelium and its relationship with immune cells are central in understanding the process through which sequential insults can cause tissue injury. Endothelial cells are obviously present in virtually every single organ and are in constant contact with immune molecular and cellular mediators. Therefore, rather than simply being a passive pipe-like structure, the endothelium serves as an important and complex immune agent and its dysfunction is closely associated with increased morbidity in SIRS and its complications via an increase of uncontrolled vascular permeability [80]. In addition, microvascular changes caused by a first hit such as hemorrhagic shock have been implicated as one of the mechanisms through which a second hit such as infection is able to cause an exaggerated systemic inflammatory response [7] that is more likely to trigger MODS/MOF.

Central to the role of the endothelium in immune responses is the glycocalyx, a thin and complex structure of proteoglycans, glycosaminoglycans, glycoproteins, and other soluble molecules, which serves as a dynamic interface through which the vascular bed communicates with the flowing blood through continuous shedding and synthesis of this layer [81, 82]. Functions of the glycocalyx vary widely, from conferring the outer surface of the endothelium an overall negative charge to regulating vascular permeability and fluidic balance, all the way to preventing erroneous and inadvertent adhesion of leukocytes and platelets to the vascular wall by mechanically shielding molecules such as intercellular adhesion molecule 1, vascular cell adhesion molecule 1, and selectins [81, 83]. The binding of cytokines in the glycocalyx also plays an important role in enclosing and effectively hiding these molecules from circulating leukocytes cell surface receptors. Loss of glycocalyx function has been observed under many inflammatory processes such as diabetes, atherosclerosis, sepsis, and IR injury due to, amongst other things, changes in the interaction of the exposed vascular bed and circulating leukocytes and increased vascular permeability [81, 82].

Endothelial cells express innate immune receptors, such as Toll-like receptors, which can trigger intracellular inflammatory responses through mediators such as MAPK and NF-*κ*B that can ultimately modulate vascular permeability and coagulation [84]. It is even conceivable that thrombi inside the microvasculature may have a role in mechanically preventing infectious agents from spreading. Albeit never actually dormant, the overt activation of the endothelium can be triggered as a natural and physiological response to the stimulation of the innate immune response via reactive oxygen signaling dominance due to an uncoupled state of the endothelial nitric oxide synthase (eNOS) or as a pivotal part of many different disease processes involved mainly in cardiovascular illnesses [85].

In healthy and undisturbed endothelium, cell junctions are constantly regulated to preserve overall vascular barrier integrity while allowing for the passage of small molecules and immune cells that are responsible for tissue surveillance [86, 87]. Activated neutrophils and their interaction with the endothelium, involving neutrophil adhesion and subsequent transendothelial migration, play a crucial role in SIRS pathophysiology and are closely related to endothelial dysfunction, associated with loss of functional intracellular contact sites. This loss of integrity of cell-to-cell contact results in tissue edema and impairment of microcirculation and ultimately leads to organ dysfunction [88–90]. Microvascular endothelium also has an integral role in postinjury priming of the innate inflammatory response. Neutrophil priming agents such as LPS and TNF cause endothelial activation by stimulating the expression of adhesion molecules (e.g., ICAM and VCAM) on the vascular bed that can account for tissue damage-driven cell migration [91–93].

The process of neutrophil activation involves complement dependent and complement independent mechanisms. When the blood comes in contact with the activated endothelium it strongly activates the complement and clotting cascades which in turn causes neutrophil activation via the anaphylatoxins C3a, C5a, and C5b9 [94], as previously discussed. Neutrophil adhesion to the endothelium is carried out by cytokines such as IL-1 and PAF but also by adenosine, prostacyclin, and cAMP. Endothelial activation causes the high expression of adhesion molecules or activation of constitutively expressed molecules like ICAM-1, leukocyte function-associated antigen-1 (LFA-1), or E-selectin [95, 96]. The endothelial activation by cytokines leads to the upregulation of adhesion molecules and can be accompanied by the expression of anaphylatoxins C3a and C5b, PAF, and LTB4. TNF-*α* and IL-1 result in neutrophil degranulation and further activation of endothelial cells. For example, neutrophil degranulation induced by TNF-*α* leads to vascular endothelium architecture destruction by proteolytic enzymes such as elastase, gelatinase, and collagenase and subsequently to the disruption of vascular wall integrity as elastase degrades the endothelial homodimeric cadherin-cadherin binding [97]. The overall result of this process is augmented permeability, adhesion, and migration of PMN and other leukocytes to locally or remotely affected tissues and organs, causing tissue damage, organ dysfunction, and ultimately MODS/MOF [91–93].

The combination of these cellular, molecular, and vascular phenomena needs to be constantly and ever so delicately balanced and regulated. The sheer simplicity of the reasoning behind the two-hit argument is that sequential and superimposed insults tend to challenge this fragile environment and are capable of synergistically rerouting the inflammatory response to a tissue injury-driven path that can be ultimately recognized through the progression of the complex SIRS/sepsis/ARDS/MODS/MOF continuum, and possibly death.

## 3. IPC: An Elegant and Ingenious Solution for Modulating the Inflammatory Response to Ischemia

As early as 1986, researches were already noticing the protective effects that short-term nonlethal ischemic cycles could have over the heart if it were to be later exposed to a prolonged, potentially fatal ischemic event [10] and in 1990 a similar phenomenon had already been identified in the brain of gerbils [98]. This basic mechanism itself can be established in a variety of different organs and systems, and the initial protection-inducing ischemia can be of varying types and profiles. IPC or ischemic preconditioning is the general term that describes such phenomena. Some aspects of preconditioning through ischemia can even be naturally occurring as it is seen in patients that suffer from cerebral transient ischemic attacks (TIAs) and do not incur in structural damage to neurons but rather are actually protected from subsequent major episodes [99] demonstrating that IPC-like mechanisms can be adaptive in nature.

In general terms, IPC can be achieved by relatively gentle and often cyclic ischemic events. A tissue that undergoes an ischemic event is more likely to survive a subsequent prolonged deprivation of oxygen. Ideal duration and proportion of these initial insults of preconditioning are species- and tissue-specific. For example IPC can be achieved in rats during one to three cycles of IR [100, 101], while in rabbits a single 5 min cycle of IR is sufficient [102] and in dogs a 2.5 min single event of IR has been proven to be effective [103].

Processes involved during reperfusion of severe ischemic injuries are a consequence of a complex sequence of events leading to changes in capillary permeability, neutrophil recruitment, complement activation, and generation of reactive oxygen species [104], similar to other inflammation-driven insults. Preconditioned systems tend to attenuate these responses to IR and ultimately ameliorate IR injury. Two distinct phases of IPC can be observed [102, 105]. An early or classic stage of protection, independent of protein syntheses, begins almost immediately after the mild ischemic insult and can be sustained for up to 3 hours [106]. A second or delayed phase lasts for up to 24 hours after the first preconditioning hit and is based on synthesis of new proteins and altered gene expression [106].

Aside from local protection of tissues close to the preconditioned area, remote or distant protection of organs (RIPC, or remote ischemic preconditioning) can also occur. In broader terms, this notion means that exposing various tissues or organs (such as limbs) to mild preconditioning ischemic events can cause other distant organs and tissues to be more resilient to following ischemic insults, even though the latter were not directly preconditioned in the first place. This had already been demonstrated in 1993 when McClanahan and colleagues showed in a preliminary report that myocardial infarct size was reduced if rabbits were previously exposed to a brief transient occlusion of the renal artery [107]. In 1996 Gho et al. proved that brief occlusion of adjacent coronary arteries, left renal artery, and anterior mesenteric artery protected the myocardium from a subsequent prolonged ischemic event [108]. A murine model subject to two hours of ischemia of the hind limb showed significant protection against local (leg skeletal muscle) and distant (intestinal injury and lung infiltrates) organ injury by a subsequent severe ischemic event [109]. IPC also caused a marked mortality decrease up to one week after ischemia [109]. Due to its effect on decreasing neutrophil infiltrates in the lung [109–111] and because of the systemic attenuation of subsequent inflammatory responses, IPC could be a tool for modulating systemic inflammation and for preventing local and distant organ injury or even SIRS/sepsis or ARDS. Another clinical application of remote or distant IPC is the protection that it delivers against acute kidney injury following major cardiac surgery, demonstrated by the reduced rate in which patients subject to the procedure demand renal replacement therapy [112]. The possibility of remotely achieving IPC is pivotal in a number of conditions. During heart surgery, for instance, the repetitive clamping and declamping of major blood vessels to induce local IPC on the heart can cause the formation of emboli in addition to the risk that short repetitive ischemic episodes can be traumatic in nature to the organ itself [106]. Therefore, the possibility of triggering remote systemic protection against ischemia in organs such as the heart is invaluable [113, 114].

The molecular mechanisms through which IPC or RIPC protection occurs are still unclear and not a single definite pathway has been established. It seems that the protective state of IPC is achieved through a combination of humoral, neural, and systemic components [106, 115]. Principle mediators are adenosine, reactive oxygen species (ROS), NF-*κ*B, bradykinin, opioids, angiotensin, endocannabinoids, and nitric oxide (NO) that alter cellular metabolism via ATP-sensitive K^+^ ion channels and receptors that direct transcription of survival proteins and activation of intracellular kinases that ultimately protect against oxidative stress [105, 115–123]. The role of the endothelium seems to be central during the development of ischemic resistance through IPC. Local and systemic endothelial function was proven to be greatly enhanced when human subjects were exposed to daily short-term limb ischemia for a period of 7 days, with measurable improvements in resting skin microcirculation and brachial artery function assessed by FMD (flow-mediated dilation), effect which lingered after the late phase of ischemic protection was over [25]. Another study has shown that IPC is capable of protecting tight junctions both functionally and structurally on hearts that would otherwise suffer from cell-to-cell collapse and edema when exposed to severe IR injury [26]. Moreover, the role of endothelial nitric oxide synthase (eNOS) and its NO output has been implicated as relevant for the ischemic preconditioning both in early and late IPC [27, 124, 125].

The reasoning behind the triggering mechanisms of IPC is that short preconditioning insults generate enough tissue damage upon reperfusion as to cause the release of adenosine (from the breaking of ATP molecules), bradykinin, and ROS [105, 126]. These substances trigger a molecular cascade that begins in the translocation and activation of protein kinase C and culminates in the phosphorylation of HSP27 via p38 MAPK and the opening of ATP-sensitive K^+^ channels [126]. The role of organelles such as mitochondria and their function in regulating intracellular Ca^2+^/K^+^ exchange has also been implicated in the mechanisms of IPC protection in neuronal tissues [127], since the artificial opening of ATP-sensitive K^+^ channels can mimic some of the effects of IPC [128]. Many molecular mechanisms of IPC have the inhibition of mPTP (mitochondrial permeability transition pore) channels as their endpoint, which, if opened, mediate cell death by ATP depletion and mitochondrial swelling [115, 129]. Activation of PKC measured by its intracellular translocation and the role of several kinases such as PI3, ERK/MAPK, and JAK/STAT are pivotal in conferring the state of protection during IPC [115, 121, 129].

Furthermore, IPC can attenuate or eliminate O_2_^−^ production and its effects by suppressing endothelin-1 (ET-1) secretion via the opening of these mitochondrial KATP channels prior to subsequent ischemic events, since ET-1 generation is related to increased production of superoxide anion and endothelial dysfunction with increased P-selectin expression and neutrophil adhesion [130].

Nevertheless, due to the complexity of these mechanisms, results generally lack consistency when it comes to defining pathways through which IPC occurs. For example, in two separate knockout models, NO produced by an increased expression of NF-*κ*B has been shown to be relevant in the ischemic protection of the heart [131], while the same role could not be demonstrated in the protection of the intestines and brain tissue [132].

The suppression of TNF-*α* and Bax (both involved in apoptotic stimulation) and the stimulation of cellular survival mechanisms such as phosphorylation of ERK-1/2 and Akt simultaneous to the upregulation of Bcl-2 have been identified in IPC of the myocardium [133] as mechanisms through which cell survival surpasses the rate of apoptosis after IR injury. Once again, results concerning the role of TNF-*α* in IPC are inconsistent. Some studies have suggested a protective self-regulatory effect that the secretion of TNF-*α* could have on the stimulation of NF-*κ*B and on the suppression of proinflammatory proteins during subsequent ischemic injury [133].

Other recently unveiled novel molecular mediators of IPC have risen to the stage as possible targets in the investigation of the protective cascades of preconditioning. A study from 2013 showed that concentrations of HIF1-*α* and procaspase-3 were higher in patients receiving RIPC by upper limb ischemia before cardiopulmonary bypass. These same patients had higher right atrial tissue and systemic concentrations of IL-1*β*, IL-8, and TNF-*α*, indicating the direct influence of RIPC in the modulation of apoptosis and inflammation [134]. The role of Cx43 (connexin 43) has also been established in both local and remote IPC, which attenuates the ischemia-induced dephosphorylation of Cx43 that would otherwise cause the mechanical, chemical, and electrical instabilities in cardiomyocytes gap junctions by opening of the Cx43 hemichannels [135, 136]. This stabilization of Cx43 might be related to its association with PKC and p38MAPK [135]. A relevant role of microRNA 144 has also recently been revealed. Levels of mRNA 144 were found to be increased after RIPC and the exogenous intravenous administration of mRNA 144 was capable of mimicking the protective effect of RIPC in rescuing tissue from IR injury. Furthermore, increased activation of Akt and p44/42MAPK and decreased levels of mTOR were observed after the administration of mRNA 144, suggesting that the molecule could serve as a biomarker for the efficacy of a conditioning procedure [115, 137].

IPC as a technique has very practical applications. It can potentially be used as therapeutic preparation for surgical procedures that require some kind of ischemic episode, like in organ transplants. Another very important application of the model for real-life pathologies is the identification of novel molecular targets that are involved in IR injury and in the protection against said injuries. The modulation of some of these molecular targets and pathways can serve as treatment for naturally occurring ischemic insults seen in strokes, in thromboembolic injuries, and in acute myocardial infarction, to name a few.

## 4. Conclusion

As stated before, neither IPC nor the multiple-hit hypotheses are particularly new ideas, but it was not until the beginning of the century that more and more research has focused on one of the two models. During this paradigm shift, the idea that the two-hit model can satisfactorily account for many real-life pathological processes has been somewhat pushed aside.

It is logical to assume that, because of its nature, IPC is a mechanism that can be spontaneously found in the body and its artificial mimicking consists of a way to tap into the underlying responses of the immune system upon inflammatory insults and can serve as a tool for modulating these responses. Therefore, the two-hit model and IPC are not competing models in principle. While the former serves as a tool to explain the intricate processes that can be brought about via multiple sequential inflammatory insults which occur during a host of disease processes, such as trauma, hemorrhagic shock, and sepsis, the latter is an active investigation of techniques and strategies that can modulate the inflammatory response to a number of ischemic insults.

Nevertheless, there is an obvious paradox surrounding the subject: how can an inflammatory, ischemic insult drive the triggering of a protective mechanism to a subsequent ischemic insult, while the two-hit model states that a priming insult, including ischemia, should prepare the body to an exaggerated response to a second hit or insult, which incidentally can also be ischemic in nature. To our knowledge, there are no definitive answers to this conundrum. That the initial preconditioning ischemic event should not be harsh enough to cause serious tissue injury while maintaining enough potency to trigger the molecular mechanisms relevant to preconditioning seems reasonable and intuitive, but, other than that, no clear differentiation has been established concerning the nature of the first event in each model. Researchers have already mapped protocols to ensure that certain ischemic events are limited in intensity and duration so that they would not serve as priming insults for the host while still holding their beneficial effects [100–103], but aside from this intuitive distinction little is known concerning what makes an insult behave in either a priming or protective manner.

During this review many pathways, examples, and applications of both the two-hit and IPC models were explored, and little juxtaposition was found. The goal behind this text was exactly that of reconciling both models and demonstrating that each of the seemingly contradictory perspectives has a lot to offer as platforms to better understand and intervene in some very real and relevant medical situations, while maintaining the fact that both theories are not mutually exclusive.
